# Carbonate-rich dendrolitic cones: insights into a modern analog for incipient microbialite formation, Little Hot Creek, Long Valley Caldera, California

**DOI:** 10.1038/s41522-017-0041-2

**Published:** 2017-11-21

**Authors:** James A. Bradley, Leslie K. Daille, Christopher B. Trivedi, Caitlin L. Bojanowski, Blake W. Stamps, Bradley S. Stevenson, Heather S. Nunn, Hope A. Johnson, Sean J. Loyd, William M. Berelson, Frank A. Corsetti, John R. Spear

**Affiliations:** 1 Department of Earth Sciences, University of Southern California, Los Angeles, CA USA; 2 Facultad de Ciencias Biológicas, Pontificia Universidad Católica de Chile, Santiago, Chile; 3 Department of Civil and Environmental Engineering, Colorado School of Mines, Golden, CO USA; 4 Soft Matter Materials Branch, Materials and Manufacturing Directorate, Air Force Research Laboratory, Wright-Patterson AFB, OH 45433 USA; 5 Department of Microbiology and Plant Biology, University of Oklahoma, Norman, OK USA; 6 Department of Biological Science, California State University, Fullerton, Fullerton, CA USA; 7 Department of Geological Sciences, California State University, Fullerton, Fullerton, CA USA

## Abstract

Ancient putative microbial structures that appear in the rock record commonly serve as evidence of early life on Earth, but the details of their formation remain unclear. The study of modern microbial mat structures can help inform the properties of their ancient counterparts, but modern mineralizing mat systems with morphological similarity to ancient structures are rare. Here, we characterize partially lithified microbial mats containing cm-scale dendrolitic coniform structures from a geothermal pool (“Cone Pool”) at Little Hot Creek, California, that if fully lithified, would resemble ancient dendrolitic structures known from the rock record. Light and electron microscopy revealed that the cm-scale ‘dendrolitic cones’ were comprised of intertwined microbial filaments and grains of calcium carbonate. The degree of mineralization (carbonate content) increased with depth in the dendrolitic cones. Sequencing of 16S rRNA gene libraries revealed that the dendrolitic cone tips were enriched in OTUs most closely related to the genera *Phormidium*, *Leptolyngbya*, and *Leptospira*, whereas mats at the base and adjacent to the dendrolitic cones were enriched in *Synechococcus*. We hypothesize that the consumption of nutrients during autotrophic and heterotrophic growth may promote movement of microbes along diffusive nutrient gradients, and thus microbialite growth. Hour-glass shaped filamentous structures present in the dendrolitic cones may have formed around photosynthetically-produced oxygen bubbles—suggesting that mineralization occurs rapidly and on timescales of the lifetime of a bubble. The dendrolitic-conical structures in Cone Pool constitute a modern analog of incipient microbialite formation by filamentous microbiota that are morphologically distinct from any structure described previously. Thus, we provide a new model system to address how microbial mats may be preserved over geological timescales.

## Paleobiology: modern microbes may tell an ancient tale

Microbial mats currently thriving in a hot pool in California may help explain the origin of fossilized evidence of early life on Earth. Modern microbial mats that are structurally similar to microbial fossil mats are rare. John Spear at the Colorado School of Mines, with co-workers from elsewhere in the USA and in Chile, examined the microbial mats growing in a geothermal pool at Little Hot Creek in California. Light microscopy and electron microscopy identified crucial fine structure similarities with branching mat structures in the fossil record. The researchers developed hypotheses to explain the influence of nutrient flow on the growth and movement of the microbes in the mats. These living mats are a useful model system to help researchers understand how ancient microbial mats formed and were preserved over geological timescales.

## Introduction

Ancient fossilized putative microbial structures appear in the rock record as morphologically distinctive indicators of early life on Earth.^1,2^ Tufts in fossilized Archaean structures have been interpreted as Cyanobacteria and thus evidence for oxygenic photosynthesis.^3^ However, as structures that have undergone lithification and post-depositional diagenetic alteration, most ancient microbialites lack preserved microfossils or unobscured information concerning their formation and the chemistry of their environments.^4,5^ An understanding of the processes that control the formation of microbialites and determining the geochemistry of these structures will help to more accurately interpret the ambient ancient environmental, geochemical and physical conditions.

Conical microbialite forms are well known from the rock record and occur at various scales (millimeter to decimeter), but modern examples, especially from carbonate-precipitating environments, are rare.^6–9^ Some examples of modern conical microbialite structures have previously been described in geothermal springs^10,11^ and the Antarctic lakes Untersee^12^ and Vanda.^13^ It is useful to characterize these and other modern conical analogs of ancient microbialites to support the development of a formation model and to better understand their preservation potential in rock over geological timescales.

**Fig. 1:**
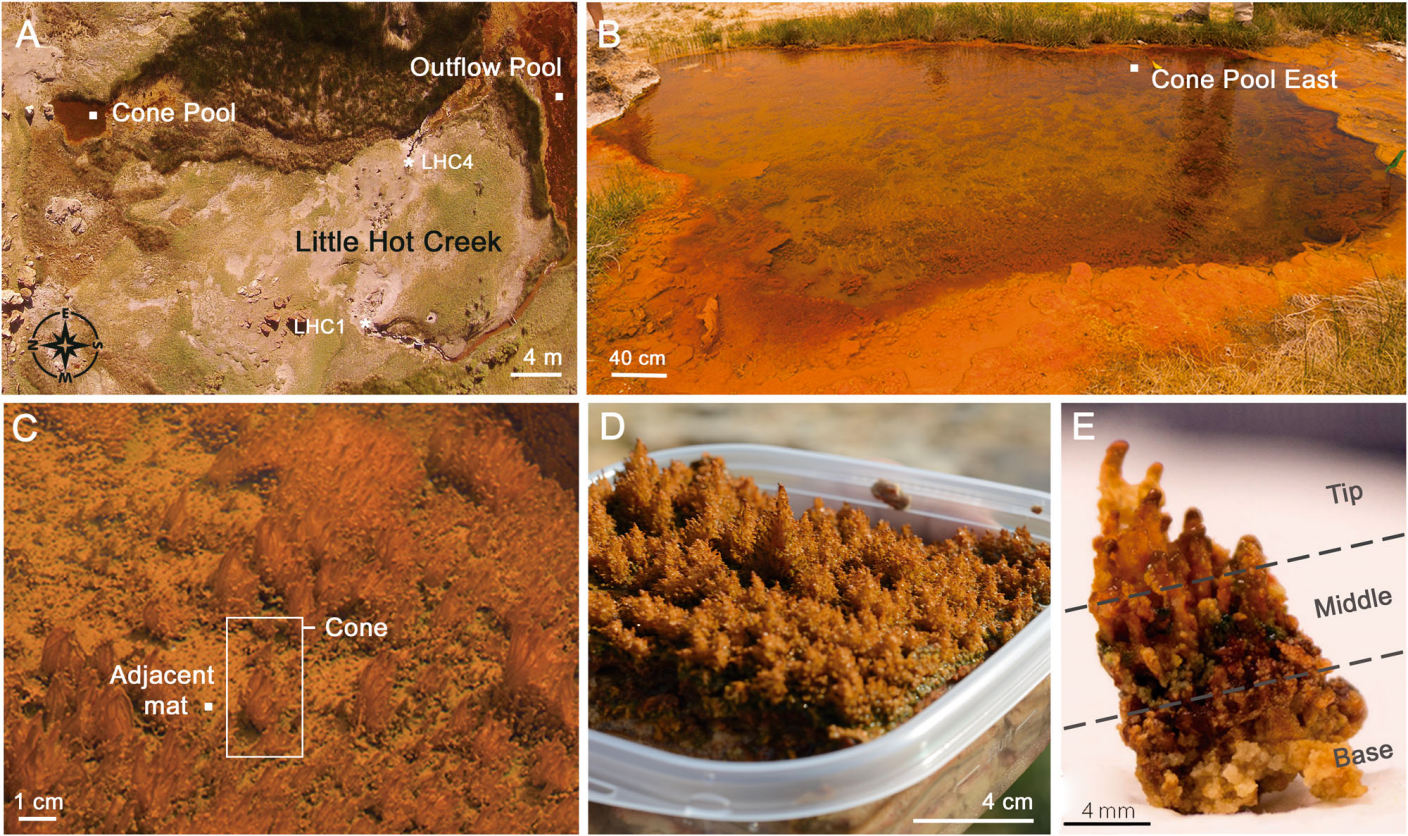
Cone Pool at Little Hot Creek. **a** Satellite view of Little Hot Creek (LHC) geothermal spring in California, USA. White squares indicate current study sampling sites. White asterisks indicate sampling sites of Vick et al. (2010). **b** View of Cone Pool from the West rim. **c** Dendrolitic cones in situ at Cone Pool East. **d** Extracted microbial mat showing dendrolitic cone assemblage and size differences. **e** Photograph of extracted dendrolitic cone indicating macrostructure. Cone Tip, Cone Middle, and Cone Base labeled

Here, we present a detailed physical, geochemical, and genomic characterization of semi-lithified carbonate, dendrolitic conical microbial structures (i.e. dendrolitic cones) from a geothermal pool (Cone Pool) at Little Hot Creek (LHC) spring, in the Long Valley Caldera, California, USA (Fig. 1). We describe the dendrolitic cone structure as a cm-scale central coniform structure on which smaller scale dendrolitic structures root, and as such, is a unique structure that has some similarity with both coniform and dendrolitic microbialites in the rock record. The dendrolitic cones, surrounding microbial mats, and water, were examined to determine their morphological structure, microbial and geochemical composition, and degree of lithification. The microbial composition of the dendrolitic cones, underlying microbial mat, and the layered microbial mat from an adjacent outflow pool (LHC Outflow pool) were determined by sequencing amplified libraries of small subunit ribosomal 16S rRNA genes. The autotrophic growth rates of the community were measured by ^13^C-bicarbonate uptake from dissected samples of the tips of dendrolitic cones. The data presented here provide a basis to better understand the structure and generation mechanism of LHC dendrolitic cones. These findings provide insight into dendrolitic cone microbialite formation and preservation in modern geothermal springs. They also provide an analog to ancient microbialites.

## Results

### Site characterization

The Cone Pool at LHC is a small geothermal pool that measures ~6 m by ~4 m and is ~0.75 m deep in the center. The pool is fed by a subsurface spring via an inlet vent along its northern rim (Fig. 1a). The geothermal spring upwelling that originates in Cone Pool trickles downstream to a second larger pool (Outflow Pool). Cone Pool and the Outflow Pool contain microbial mats, yet, uniquely, Cone Pool contains partially lithified dendrolitic cones that emerge from an underlying, laminated microbial mat foundation. Physical and chemical characteristics of Cone Pool and the Outflow Pool spring water are presented in Table 1. At the time of sampling, Cone Pool was substantially warmer (45.6 °C) than the downstream LHC Outflow Pool (34.1 °C). Cone Pool and the Outflow Pool exhibited similar slightly alkaline pH (8.08 and 8.29 respectively), and both were super-saturated with respect to calcite (Ω_Ca_ = 4.08 and 3.40 respectively) (Figure S1). Concentrations of dissolved total CO_2_ (TCO_2_), dissolved oxygen, and Ca^2+^, K^+^, Mg^2+^, and Na^+^ were similar between the Cone Pool and Outflow Pool (Table 1). Based on visits to Cone Pool prior to and after the sampling trip of the present study (June 2015), dendrolitic cones may be an ephemeral feature of the microbial mat: dendrolitic cones were visible from the first visit (December 2013) until September 2015, however, were not visible on return to the site in June 2016.

**Table 1:** Physical and chemical properties of stream water at cone pool and outflow pool

Site	Temperature^a^	Ω	TCO_2_^b^	pH	Dissolved oxygen^c^	Ca^b^	K^d^	Mg^b^	Na^b^
Cone Pool	45.6	4.08	11.98	8.08	130.3	0.43	0.70	29.42	17.49
Outflow Pool	34.1	3.40	11.52	8.29	141.4	0.26	0.75	35.76	18.23

^a^ °C

^b^ Millimolar (mM)

^c^ Percent relative to vapor saturated air (100% DO)

^d^ Micromolar (μM)

### Dendrolitic cone description

The dendrolitic cones ranged in height between 0.5–2.0 cm (Fig. 1c) and were orange to brown in color, with an upright, fractal, branching appearance; that is, the cm-scale dendrolitic cones had mm-scale protrusions upon them, creating an ‘arborescent’ appearance. The mm-scale protrusions were somewhat rounded at the tip, and oriented at an upward angle from the surface of the dendrolitic cone. The dendrolitic cones were rigid, did not deform when removed from the pool, and remained firm to touch upon collection and transport (Fig. 1d, e). Geochemical analyses revealed that the dendrolitic cones were predominantly comprised of CaCO_3_ (76.1% dry weight). Organic carbon comprised 5.9% of the dry weight of dendrolitic cone material. One representative dendrolitic cone, measuring 1.2 cm across the base and 2 cm high, was subdivided into Tip, Middle, and Base for further analysis (Fig. 1e).

**Fig. 2:**
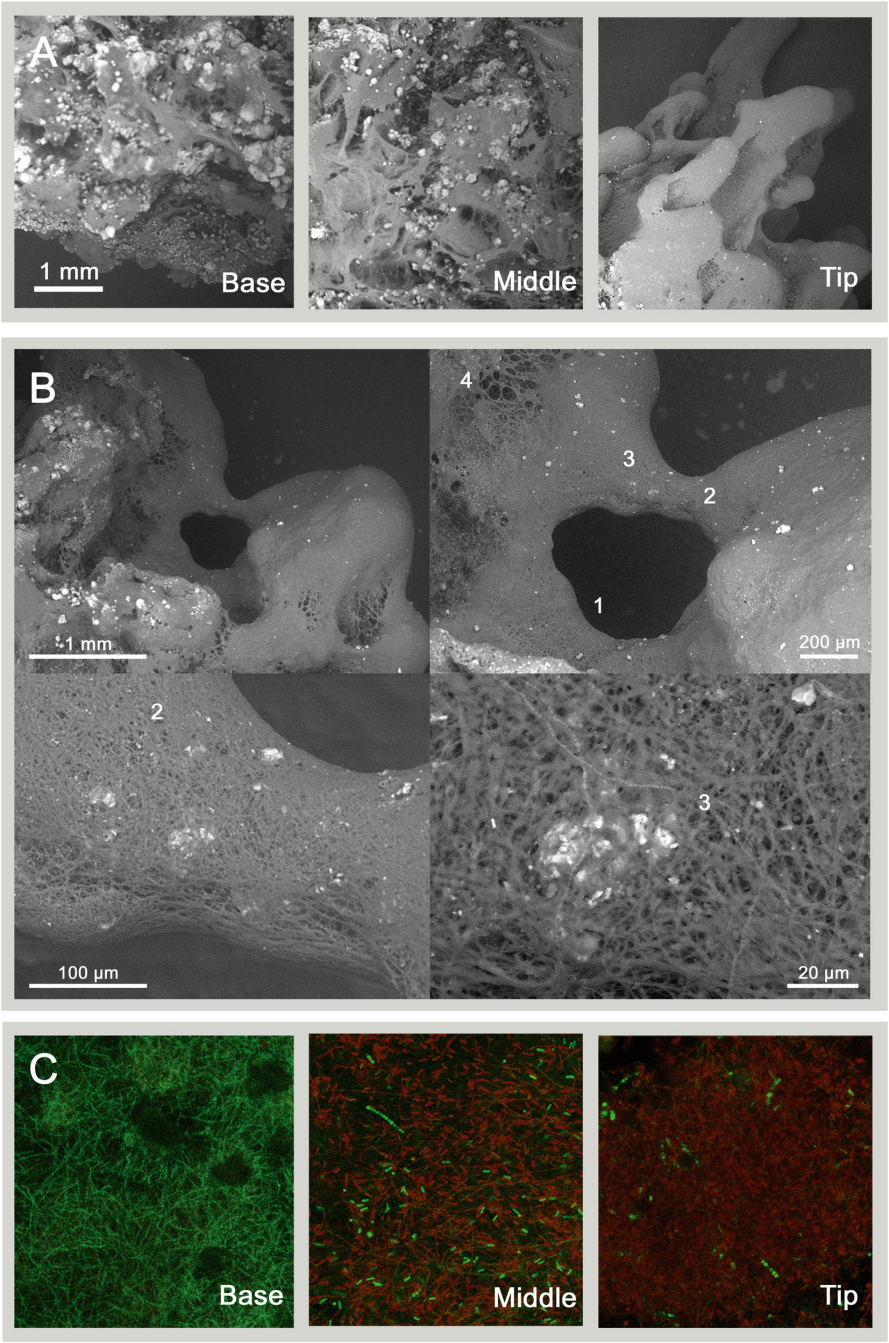
Microscopic characterization of dendrolitic cones. Scanning electron microscopy images of **a** dendrolitic cone macrostructure and **b** microscale structures. Notable features of dendrolitic cone micro-structure indicated by numbers: (1) major voids, (2) bridging structure, (3) CaCO_3_ grains, (4) small ‘bubble-like’ voids. **c** Confocal microscopy images of dendrolitic cone filament sections, at a magnification of ×400. Photoautotrophic cells are shown in red

**Fig. 3:**
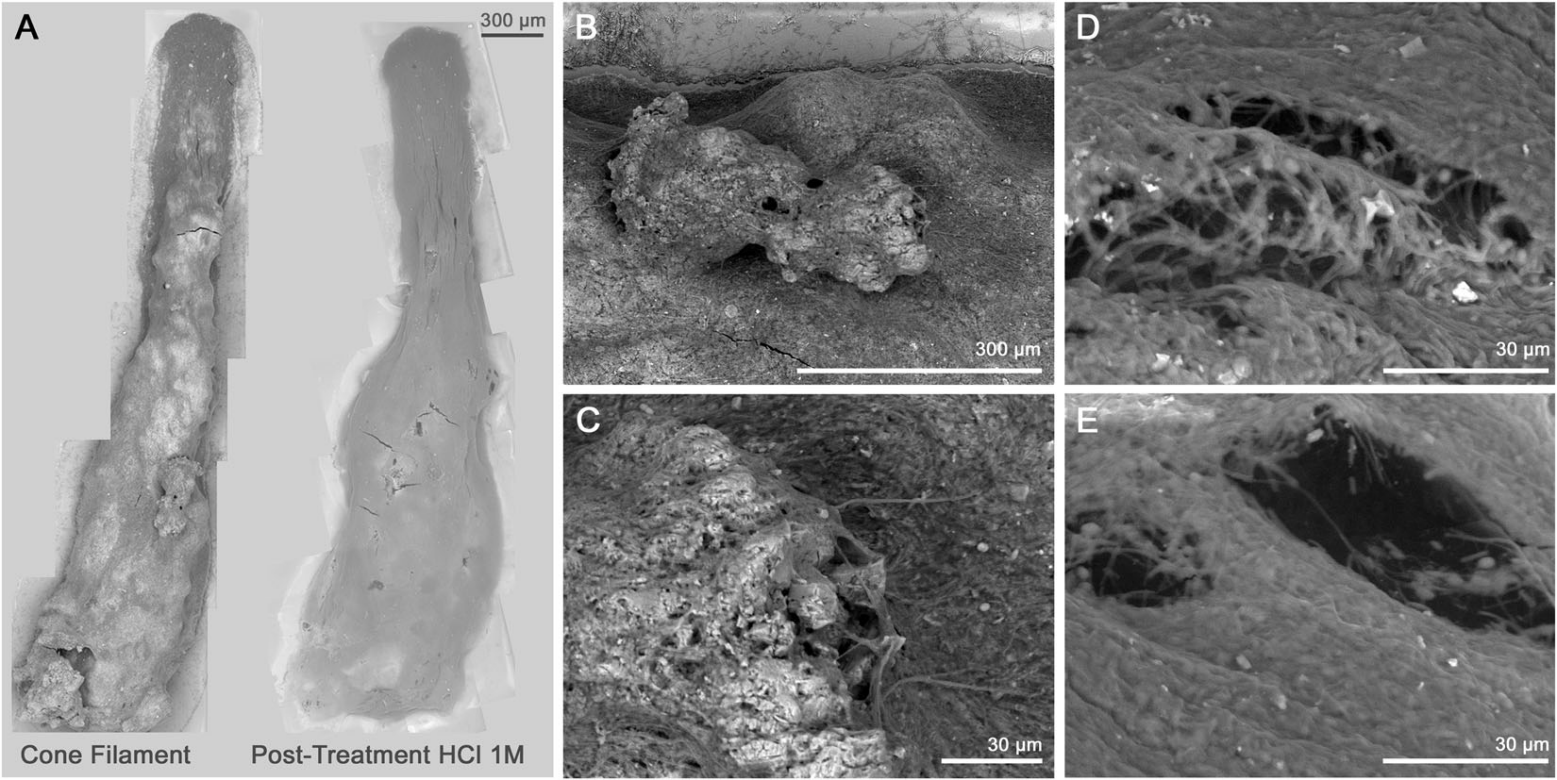
Analysis of structure of dendrolitic cone filament. **a** Scanning electron microscopy images from an individual dendrolitic cone filament, pre-(left) and post-(right) treatment with one drop of 1 M HCl to remove calcium carbonate. **b**, **c** Grains intertwined between filamentous microorganisms, prior to treatment with 1 M HCl. **d**, **e** Post-treatment observation of voids

Scanning Electron Microscopy (SEM) imaging revealed that the Cone Tip, Middle, and Base contained morphologically distinct microstructures. The dendrolitic cone tip was covered with the mm-scale protrusions comprised of domed pinnacles (0.5–1 mm length) (Fig. 2a). The pinnacle elements consisted of intertwined microbial filaments that formed matrices and bridging structures. Voids in the dendrolitic cone on the scale of >600 μm diameter occurred between densely packed filamentous bridging structures (Fig. 2b). These filaments appeared to be intertwined around calcium carbonate grains ranging in size from ~1–50 μm in diameter, which were slightly rounded but contained multiple facets. The Cone Middle exhibited a web of intertwined filaments with increasingly abundant mineral grains (white) compared to the Cone Tip (Fig. 2a). Examination of the Cone Base revealed an even higher degree of mineralization: grains in the Cone Base were largest and most common (Fig. 2a, Base). The high degree of mineralization in the Cone Base likely occluded the microbial filament structures that characterized the upper parts of the dendrolitic cone. A notable but uncommon feature from SEM imaging was the appearance of hour-glass shaped void structures (Fig. 2b) that, at ~50 μm diameter, were substantially smaller than the larger voids (>600 μm) mentioned above. These smaller voids seemed to cluster together in spatially discrete locations, where filaments were less tightly bundled. Confocal analyses showed that the auto-fluorescence in the dendrolitic cone increases from the Cone Base to the Cone Tip, suggesting a greater abundance of photoautotrophic microorganisms in upper sections of the dendrolitic cone (Fig. 2c). SEM images of a dendrolitic cone sample were taken before and after the addition of 1 M hydrochloric acid (HCl), which resulted in the dissolution of the calcium carbonate grains, leaving voids (~50 μm) in the sample (Fig. 3). In addition, the sample lost its rigidity.

### Growth of microorganisms in the Cone Tip

The uptake of ^13^C-labeled bicarbonate was measured in samples from the Cone Tip and the microbial mat from the outflow pool. Fixation of bicarbonate in samples from the Cone Tip was confirmed with measured rates of autotrophic growth of 0.15% d^−1^ (±0.01; 1 SD). Autotrophic growth in the microbial mat from the Outflow Pool was 0.17% d^−1^ (±0.05; 1 SD). Thus, measured growth relative to killed controls showed that resident autotrophs were actively fixing inorganic carbon in the dendrolitic cone tips and in the microbial mat in the Outflow Pool.

### Microbial community assemblage in mats at Little Hot Creek

**Fig. 4:**
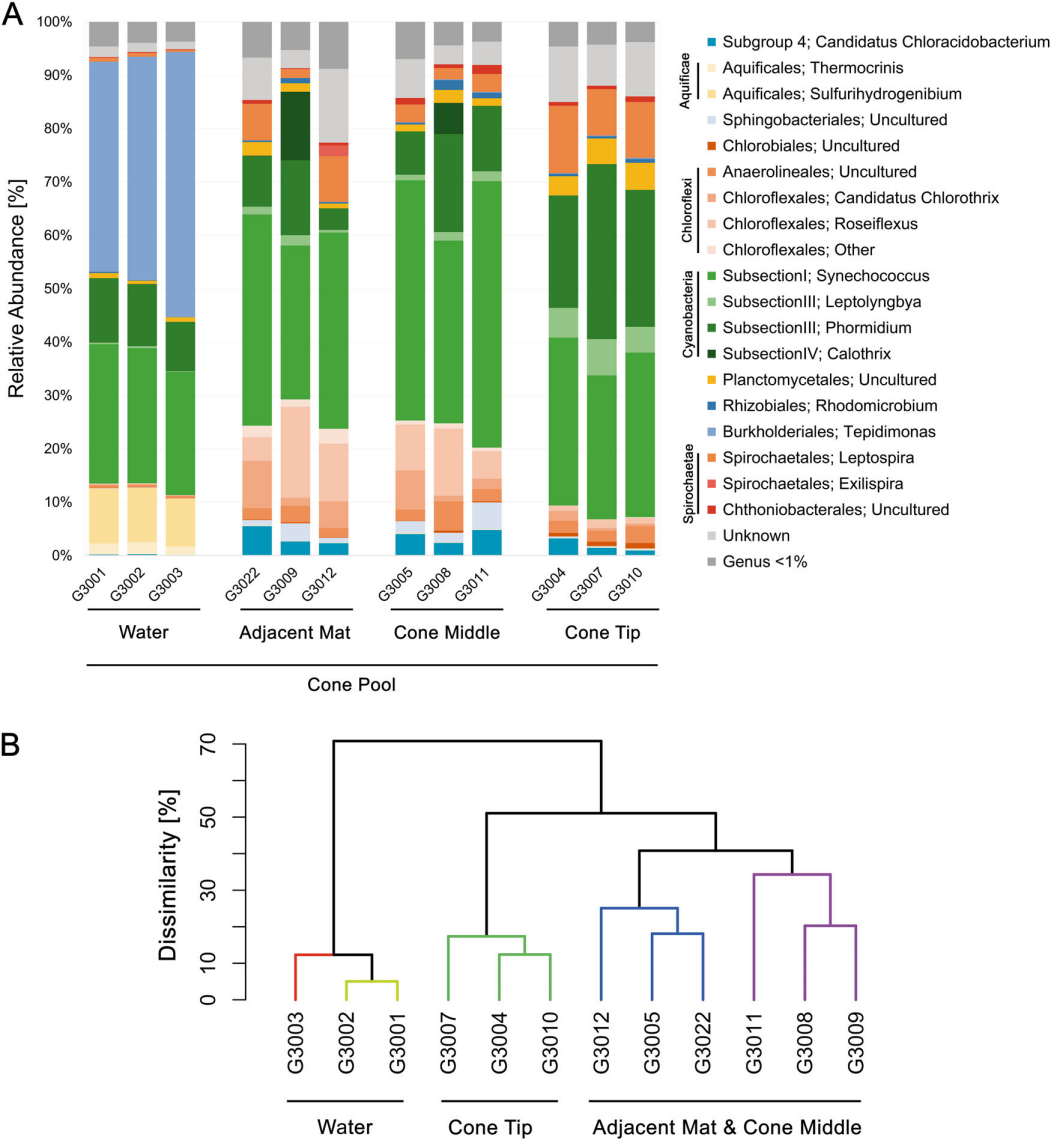
Bacterial community composition from Cone Pool East. **a** Relative abundance plots of different sections of the dendrolitic cones, including the surrounding bacterial communities present in the Adjacent Mat and Water. Analysis was performed using 16S rRNA gene sequencing. **b** Cluster dendrogram between communities from Cone Pool East, using Bray-Curtis dissimilarity and SIMPROF test. Significant clustering (*p* < 0.05) is indicated with colored branches

Several Operational Taxonomic Units OTUs were more abundant in the Cone Tip compared to the Cone Middle. These included genera most closely related to the Cyanobacteria *Leptolyngbya* (1–6%), and *Phormidium* (~13–26%), an uncultured Planctomycetes (~1–5%), and the Spirochaetae genus *Leptospira* (~3–11%). Lower relative abundances in members of the Chloroflexi (~16–5%), and Bacteriodetes (~3–0.2%) were noted in the Cone Tip compared to the Cone Middle, along with a marked decrease in members of the Cyanobacteria genus *Synechococcus* (~43–30%). Comparative community analyses based on 16S rRNA gene library sequencing of dendrolitic cone subsections and the Adjacent Mat (top layer of mat adjacent to growing dendrolitic cones) showed remarkably similar microbial communities (Fig. 4a). Although no statistically significant difference was found between samples from dendrolitic cone subsections and the Adjacent Mat using *β*-diversity analyses (data not shown), a dissimilarity analysis was performed to determine if communities were different across the dendrolitic cone structure. Cluster dendogram representation showed that samples from Cone Tip were different from surrounding water, Cone Middle and Adjacent Mat (Fig. 4b). Branches of the same color in the analysis represent significant clustering evaluated with the SIMPROF test. This distinctive shift in community composition of Cone Tip suggests that microbial populations with higher abundance of photosynthetic, phototrophic and filamentous bacteria may play a role in formation or stabilization of the dendrolitic cone structure.

**Fig. 5:**
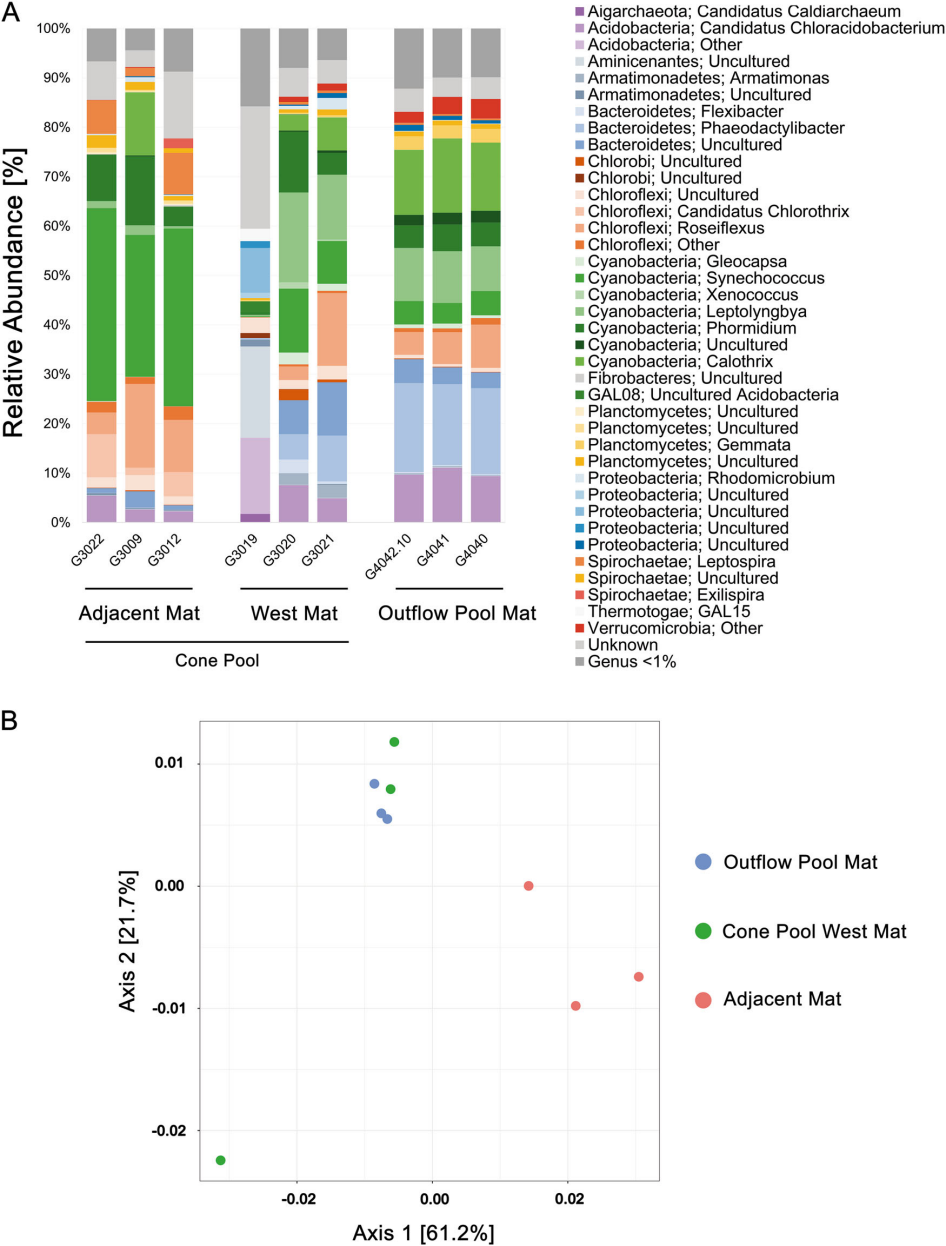
Bacterial community composition of mats from Little Hot Creek. **a** Relative abundance plots of mats based on 16S rRNA gene sequencing from the Outflow Pool, Cone Pool West and Cone Pool East adjacent to the dendrolitic cones (Adjacent Mat). **b** Principal coordinate analysis (PCoA) of variance between communities from mats in LHC

Analysis of 16S rRNA gene libraries showed that similar relative abundances of members of the Phyla Bacteroidetes, Chloroflexi, and Cyanobacteria (~19, ~10, and ~40% respectively) were found in the top layer of the Outflow Pool microbial mat and the top layer of mat from the west side of Cone Pool (Cone Pool West) (Fig. 5a). When compared to the Adjacent Mat, the relative abundance of Bacteroidetes decreased (approximately −17% decrease to ~2%), but the Chloroflexi (approximately +10% to ~20%) and Cyanobacteria (approximately +10% to ~50%) increased (Fig. 5a). Values presented here are averages of relative abundance percentages from triplicate samples.

The microbial communities from each sample type were compared via *β*-diversity analyses and visualized using principal coordinate analysis (PCoA) (Fig. 5b). While the communities between the Outflow Pool, Cone Pool West, and Adjacent Mat appear similar in composition (Fig. 5a), Adjacent Mat samples showed a distinct increase in Cyanobacteria (the genus *Synechococcus* specifically; approximately +30% shift) and therefore, these samples were separated from the rest in the ordination (Fig. 5b).

## Discussion

The structure of the dendrolitic cones present in Cone Pool are morphologically distinct from any other modern-day conical or dendrolitic microbialite structures previously described in the literature. Notably, the dendrolitic cones lacked laminations, distinguishing them from laminated coniform stromatolites described at Antarctic lakes Untersee and Vanda,^12,13^ and Yellowstone National Park geothermal spring-associated mats and stromatolites.^6,10,14,15^ The dendrolitic cone structures present in Cone Pool were also visually and morphologically different from preserved Archean and Proterozoic conical stromatolites,^1^ which are laminated and do not show fractal-like branching fabrics. However, the distinctly arborescent appearance of the dendrolitic cones resembled a hybrid of modern-day pinnacles^12^ and ancient dendrolites/shrubs.^16,17^ A particularly striking difference between the dendrolitic cones present in Cone Pool and those found elsewhere in modern settings was that they were partially lithified, appearing firm and crunchy (Fig. 2) rather than soft and spongy.^12,13,15^ The crunchy nature of the dendrolitic cones, comprised predominantly of CaCO_3_ (76.1% dry weight), is relevant to the potential preservation of these structures and thus their use as a paleoenvironmental analog. The high degree of mineralization may have permitted the apparent preservation of macro-structure and micro-structures following sampling, transportation, and storage. However, SEM images presented here are not of sufficient magnification to determine the degree of lithification of individual microbial filaments. Higher magnification images of individual microbial filaments may enable this supposition to be reconciled, and this should be a priority in future studies.

SEM images clearly showed that the dendrolitic cones were comprised of microbial filaments. These filaments, in combination with calcium carbonate minerals, formed what appeared to resemble bridging structures, which likely provide structural support for the dendrolitic cone form (Fig. 2b). These bridging structures are not unlike what has been seen in the microbial component of certain speleothems described in a geothermal mine adit.^18^ The microbial filaments comprising the dendrolitic cones of Cone Pool had no well-defined, consistent spatial orientation. Instead, the microbial filaments appeared to be intertwined around calcium carbonate grains (Figs. 2 and 3). Intertwined filamentous dendrolitic cones at LHC were remarkably different from modern siliceous stromatolites from Obsidian Pool Prime, Yellowstone,^6–8,19^ where filaments within the same lamination were oriented in a uniform direction. In both cases, however, the interlocking organization of filaments at Cone Pool and Obsidian Pool Prime may give rise to firm, microbiotically built, physically stable macrostructures. This contrasts with the soft, non-lithified, pinnacle-like structures identified previously in both Yellowstone^15^ and Antarctica.^12,13^ It has been suggested that physical entanglement of filaments generally increases mat cohesion and thus aids the development of such complex microbialite structures^20,21^ and perhaps leads to increased preservation potential.

### Hourglass structures

SEM imaging revealed hour-glass shaped structures approximately ~50 μm in diameter defined by bundles of filaments surrounding voids (Fig. 2b). These structures are remarkably similar in size and morphology to structures preserved in a laminated siliceous stromatolite from a Yellowstone geothermal spring,^8^ which have been interpreted as relics of oxygen-rich bubbles generated during microbial photosynthesis. The high relative abundance of members of the phylum Cyanobacteria combined with an increased abundance of members of the phylum Planctomycetes in the Cone Tip could be related to the hour-glass structures. It has been found that the genus *Isosphaera*, a strict aerobic organism, can harbor gas vesicles that may help to maintain the buoyancy of cells in warm water conditions.^22^ This has also been described for benthic Cyanobacteria, which have been shown to form bubbles derived from photosynthetic activity.^23^ These bubbles may be preserved within mats upon CaCO_3_ precipitation (Fig. 2b), similar to the model described by Bosak et al. (2010)^24^ for photosynthetic mats in Yellowstone. The preservation of hour-glass structures in dendrolitic cones at LHC (and similar to stromatolites at Yellowstone) suggests that the rate of mineralization of bacterial filaments is on the timescale of the lifespan of a bubble, perhaps as short as a diel cycle. Oxygen production and bubble-forming potential is highest during daylight hours, while gas dissolution/utilization occurs during nighttime hours. In the Obsidian Pool Prime example of stromatolites in Yellowstone,^8^ rapid silicification of filaments occurred as hydrothermal fluids rich in dissolved silica cooled near the pool margins, preserving the hourglass structures likely surrounding oxygen bubbles. Similarly, the Cone Pool hourglass structures are preserved, but with calcium carbonate. Fresh geochemical constituents are continuously delivered from the spring source.

### Carbonate precipitation and dendrolitic cone lithification

The Cone Pool water was supersaturated with calcium carbonate (Ω_Ca_ = 4.08). Images prior to and after acidification with HCl show how a loss of structural integrity was caused by the dissolution of CaCO_3_ grains that were previously bound between inter-twined microbial filaments (Figs. 3b, c, d and e). This indicates a direct link between the presence of carbonate grains and the structural integrity of dendrolitic cones, and implies that mineralization of the structure is responsible for the resistance to deformation upon removal from the pool. It was difficult to determine unambiguously if the microbial community played a major role in the precipitation of minerals, for example through alteration of the micro-environment via metabolic processes, or enzymatic activation. However, mineralization increased with depth in the dendrolitic cones (Fig. 2a), suggesting progressive precipitation of CaCO_3_. Photosynthetic, chemosynthetic, thermophilic and heterotrophic microorganisms can induce biofilm calcification by actively mediating the formation of calcium carbonate in aquatic environments,^25,26^ and may be critical in mediating the formation of dendrolitic conical microbialite structures.^27^ Results from ^13^C incorporation experiments suggested that photo-autotrophy was an active metabolism for microbes in Cone Pool. We detected an increase in organic matter carbon by 0.15% per day through uptake of inorganic bicarbonate. The relative higher abundance of auto-fluorescent pigments in the Cone Tip and Middle compared to the Cone Base (Fig. 2c) suggested an abundance of autotrophic microorganisms in the upper sections of the pinnacles. Photosynthetic activity in the Cone Tip may promote carbonate precipitation by removing CO_2_ from the spring water and increasing alkalinity (Equation 1):

1 $$106\,{\rm{CO}}_2 + 16\,{\rm{NO}}_3^ - + {\rm{HPO}}_4^{2 - } + 122\,{\rm{H}}_2{\rm{O}}\,+ \\ 18\,{\rm{H}}^ + \to {\rm{C}}_{106}{\rm{N}}_{16}{\rm{H}}_{263}{\rm{O}}_{110}{\rm{P}} + 138\,{\rm{O}}_2$$

Although this equation is a gross simplification of photosynthetic carbon fixation reactions occurring in the mat, the saturation state of CaCO_3_ in the spring water is highly sensitive to changes in CO_2_ concentration and alkalinity (Figure S1). Thus, photosynthetic activity in the dendrolitic cone may drive localized concentration gradients in water chemistry and encourage carbonate precipitation, driving increases in dendrolitic cone firmness. Microbial filaments and their extra-cellular secretions can also mediate crystal nucleation in geothermal springs by acting as nuclei upon which mineral growth may proceed.^28,29^ Quantitative characterization of extracellular polymeric substances (EPS) in dendrolitic cone sections would provide additional insight on the role of EPS as nucleation points for carbonate precipitation, and we suggest this as a focus of future investigations into carbonate-based microbialite formations. Further, the accumulation of calcium carbonate in the base of the dendrolitic cones could also result from secondary processes associated with the heterotrophic metabolisms of the microbial community in the dendrolitic cone base. Calcium carbonate precipitation could be favored in environments rich in organic matter, by ammonification, dissimilatory nitrate reduction, degradation of urea or uric acid and sulfate reduction. Production of CO_3_^2−^, HCO_3_^−^, and ammonia (for nitrogen metabolisms) or hydrogen sulfide (for sulfate reduction) may cause a pH change that induces carbonate precipitation.^30^ The relative importance of individual microbial metabolisms on dendrolitic cone mineralization warrants further attention.

### Model of dendrolitic cone formation

Our δ^13^C data from the growth experiment shows that microorganisms in the Cone Tip are actively fixing dissolved inorganic carbon. The similarity in autotrophic growth rate between the Cone Tip (0.15% d^−1^) and the microbial mat in the Outflow Pool (0.17% d^−1^) indicated that growth differences are not driving dendrolitic cone formation. However, in a low-flow environment, such as between filaments within the dendrolitic cones, the development of diffusive gradients on a micro-scale may promote the movement and migration of microbes. Experimental evidence has suggested that slow diffusive transport in stagnant fluids in-between microbial filaments causes limited exchange of nutrients and promotes a growth response in modern cone-forming microbial communities.^11^

The distinct morphological features of the dendrolitic cones present in Cone Pool suggest that biological activity may exert a control on the formation of these structures. The observed differences between the microbial communities found in the Cone Tip and other locations, based on 16S rRNA gene sequence analysis, support the hypothesis that biological activity may be a controlling factor on dendrolitic cone formation and lithification. Our data show that filamentous organisms with the potential for photo- and chemo- taxis are more abundant in the tip of the dendrolitic cone than in the Adjacent Mat. OTUs most closely related to the Cyanobacterial genus *Leptolyngbya* were more abundant in the Cone Tip relative to the Cone Middle and Adjacent Mat. Members of this same genus were abundant in coniform mat structures in Yellowstone geothermal springs and other incipient microbialite structures in aqueous environments.^10,12,14,20,31^ Furthermore, an OTU in the Cone Pool (between 1–10% relative abundance) most closely related to *Gloeomargarita lithophora* (Figure S2, OTU 325) was identified. Previous research has shown it to be capable of internal biomineralization of carbonate grains.^32^ More recently Benzerara et al. (2014)^33^ found that internal biomineralization of carbonate was much more common in cyanobacteria than previously thought, including some strains of *Synechococcus*. While we have no evidence of this type of biomineralization in the Cone Pool system, the omnipresence of *Synechococcus* may allude to this type of process. The potential role of internal mineralization on microbialite formation warrants further investigation in future studies. Moreover, members of the Planctomycetes genus *Isosphaera*, a non-Cyanobacterial organism with phototactic capabilities,^34^ was found in low abundance (Table S21, OTU 114). Bacteroidetes, which are present in 16S rRNA sequences throughout LHC, are present in several other lithifying microbialite systems.^35,36^ These organisms are known to be able to degrade high molecular weight organic matter and biopolymers^37,38^ and are often found, as they are at Cone Pool, in close relation to Cyanobacteria and other primary producers.^35,36^ The possible symbiotic relationship between primary producers such as Cyanobacteria and heterotrophic Bacteroidetes at Cone Pool warrants further exploration, since these groups may contribute to biologically induced lithification.^30^

### Relevance to ancient microbialites

Unlike the conical incipient stromatolites found in Lake Untersee, Antarctica,^12^ and modern stromatolites from Obsidian Pool Prime, Yellowstone,^6–8,19^ the dendrolitic cones in Cone Pool are not laminated, and thus comparisons with the most common conical stromatolites (e.g., *Conophyton*) is not warranted. However, the surface expression of the cm-scale dendrolitic cones with mm-scale protrusions does appear to resemble fractal-like microbial fabrics from the geologic record, e.g. carbonate shrubs and/or dendrolites^16,17^ (Fig. 1e). Interestingly, the formation of carbonate shrubs/dendrolites is commonly attributed to calcimicrobes of unknown affinity, or coccoidal bacteria of some kind.^39^ The Cone Pool examples demonstrate that shrub-like morphologies can originate from filamentous microbes as well. These incipient microbialite dendrolitic cones may constitute a modern analog to address how complex microbialite structures are formed, become part of the rock record, and also inform subsequent interpretation of paleoenvironmental conditions and biological evolution through fossilized stromatolites and microbialites.

## Methods

### Study site

The Cone Pool is part of the LHC geothermal spring system located within the Long Valley Caldera, Mono County, California, USA (37°41’N, 118°50’W). The site has been described previously in Vick et al (2010).^40^ LHC is in a mixed geological setting of Holocene alluvium silts, sands and gravel, lacustrine sediments, and Pleistocene sandstone and conglomerates. The Cone Pool is slightly northeast of the accompanying geothermal spring outlet in that same area.

### Sampling

All samples and measurements were taken on 26th June 2015. Samples of each dendrolitic cone were collected for molecular and microscopic analyses using sterile scalpels (Sklar Surgical Instruments, West Chester, PA, USA). Each dendrolitic cone was sectioned into the Cone Tip, Cone Middle, Cone Base, and Adjacent Mat (Fig. 1). Each sub-sample was bifurcated, where half were preserved for microscopy, and half were used for DNA extraction. Samples used for DNA extraction were placed in ZR BashingBead™ (Zymo Research Corp., Irvine, CA, USA) lysis tubes containing 750 µL of *Xpedition*™ (Zymo Research Corp., Irvine, CA, USA) Lysis/Stabilization Solution, and homogenized for 45 seconds in the field. For molecular analyses of the surrounding water community, approximately 2.4 L of spring water was filtered through triplicate 25 mm 0.22 µm polyethersulfone (PES) filters (Merck Millipore Corp., Darmstadt, Germany). Each filter was removed aseptically and transferred to ZR BashingBead™ lysis tubes and preserved as above.

Fluid samples for water chemistry were taken in triplicate by drawing up approximately 12 mL of spring water from the center of the stream closest to the station marker, and then filtering it through a 25 mm 0.45 µm PES filter (Merck Millipore Corp., Darmstadt, Germany) into 15 mL polypropylene conical tubes (Corning, Inc., Corning, NY, USA). A volume of 4 ml of filtered spring water was added to pre-weighed, evacuated Exetainer® (Labco Limited, Lampeter, Ceredigion, UK) vials for TCO_2_, and 2 dram vials (Fisher Scientific, Waltham, MA, USA) were overfilled and capped for cation analyses. All samples were stored on ice and transported for further lab analyses.

Samples for microscopic analyses were transported on ice, and refrigerated for 4 days before confocal microscopy analysis. Samples were then imaged by SEM 10 days later (a total of 14 days after sampling).

### Water chemistry

Water temperature, pH and oxygen concentration were measured using a SevenGo Duo pro^TM^ (Mettler-Toledo, LLC, Columbus, OH, USA). Exetainer® vials containing water samples for TCO_2_ concentration analyses were weighed and the vial masses subtracted to determine water volume. Fluids were then acidified with 10% phosphoric acid using an Automate® Carbonate Preparation device (Automate FX, Inc., Bushnell, FL, USA) in order to liberate all inorganic carbon as gaseous CO_2_. Produced CO_2_ was passed into a Picarro® G-2121i Cavity Ringdown Spectrometer (CRDS) (Picarro Inc., Santa Clara, CA, USA) and the TCO_2_ content determined by comparison with NBS 915b pure carbonate standards. The saturation state of stream water from Cone Pool and the Outflow Pool with respect to calcium carbonate (Ω_Ca_) was calculated using measured calcium ion concentrations, temperature, a saturation product (*K*_sp_) of 9.81 × 10^−9^, and PHREEQC modeling software.^41^

### Inorganic and organic carbon, and ^13^C bicarbonate uptake

Samples of individual dendrolitic cones with the underlying mat from Cone Pool, and the microbial mat in the Outflow Pool, were cored with a 10 mm diameter plastic drinking straw and placed into individual test tubes (~25 ml). Inorganic C content of dendrolitic cones was determined by first drying portions of the dendrolitic cone, grinding and weighing 20–40 mg into an Exetainer® vial with a septum cap. Tubes were evacuated a vacuum pump, acidified with 10% phosphoric acid and the resultant CO_2_ analyzed using Picarro® G-2121i CRDS (Picarro Inc., Santa Clara, CA, USA); specific methods are described in Subhas et al. (2015).^42^ Reagent grade CaCO_3_ served as a standard. Organic C content of dendrolitic cones was determined by the difference between Total C and Inorganic C (weight %). Total C was determined by weighing 2–10 mg of dried powdered dendrolitic cone material into an aluminum-foil cup and burning with O_2_ in a Costech Elemental Analyzer. The CO_2_ produced in combustion was determined quantitatively by the Picarro method (above). An internal standard of San Pedro Channel mud (San Pedro, California) was used to calibrate the % C.

For growth rate experiments, 0.22 µm filtered spring water from Cone Pool was amended with ^13^C labeled sodium bicarbonate to a final concentration of 4–5 mM and δ^13^C of 1800 per mil. The spring water was then added to 10 ml test tubes to fill completely without headspace and closed with a rubber stopper. Six different dendrolitic cones were incubated with and without the addition of 100 µL saturated mercury chloride + 300 mM sodium azide as an abiotic control. The vials were incubated at 40 °C for 24 h in a 12 h light-dark cycle. Following the incubation, the top one third of the dendrolitic cone (excluding any mat material) was removed and acidified with 1 M HCl to dissolve calcium carbonate. The sample was then centrifuged at 3000 × *g* for 5 min in 15 ml tubes and washed with PBS buffer three times to remove all inorganic carbon. The resulting biological pellets were dried overnight at 60 °C, ground in an agate mortar and pestle, weighed to 2–3 mg, and then packaged into aluminum foil cups. As above, the ^13^C/^12^C ratio of control and experimental samples were determined by Picarro analysis. An isotope mass balance (using ^13^C/^12^C ratios) was constructed to establish the amount of spiked bicarbonate uptake into the biomass. Replicate measurements provide confidence of precision to ± 0.01 per mil (1 SD). In all cases, the poisoned material was of unchanged isotopic value and the non-poisoned organic carbon was isotopically heavier. In all analyses, standards were run that relate the amount of organic carbon to the ppm of CO_2_ determined by the Picarro® G-2121i CRDS and the standard curve was linear over a range of 8000 p.p.m. CO_2_ with *R* ^2^ > 0.999. The calibration of carbon isotopes, standardized using U.S. Geological Survey (USGS) 40 standard material, yielded an uncertainty of <0.1 per mil through a range of 1500 to 7500 p.p.m. CO_2_. All samples delivered CO_2_ within the range of standards. Rates of ^13^C uptake were converted to an estimated rate of autotrophic growth.

### Microscopy and morphological characterization

For confocal microscopy, a dendrolitic cone dissected according to Fig. 1e was used. Wet samples were mounted onto clean glass slides, covered with square cover slips, and sealed with nail polish. Samples were observed at ×400 magnification on a Leica TCS SP2 Inverted Scanning Confocal Microscope (Leica Microsystems Heidelberg GmbH, Heidelberg, Germany). SEM microscopy was performed using both a fully dissected dendrolitic cone to determine structure and a full dendrolitic cone filament to analyze the contribution of minerals to the rigid structure and appearance of the dendrolitic cone. Samples were mounted onto an aluminum stage and observed with a Hitachi TM-1000 environmental SEM (Hitachi Ltd., Japan). Imaging was performed prior to and post acidification with 1 M HCl.

### DNA extraction and 16S rRNA gene library sequencing

DNA extraction was performed using an *Xpedition*™ Soil/Fecal DNA MiniPrep kit according to manufacturer’s instructions (Zymo Research Corp., Irvine, CA, USA). Extracted DNA was amplified using primers that spanned the V4 region of the 16S rRNA gene between positions 519 and 802 (*Escherichia coli* numbering), producing a product of approximately 266 bp. The primer pair represents a broad distribution of both the Bacteria and Archaea.^43^ The forward primer (M13L-519F: 5′- **GTA AAA CGA CGG CCA G**CA CMG CCG CGG TAA -3′) contains the M13 forward primer (in bold), followed by the 16S rRNA gene-specific sequence (underlined) to allow for barcoding of each sample in a separate reaction.^44^ The reverse primer (785 R: 5′-TAC NVG GGT ATC TAA TCC-3′) was taken directly from the reverse primer “S-D-Bact07850b-A-18” in Klindworth et al.^43^

Each 50 µL reaction mixture consisted of: 1 × 5 PRIME HOT master mix (5 PRIME Inc., Gaithersburg, MD), 0.2 µM of each primer, and molecular grade water. A volume of 4 µL of extracted template DNA was added to each reaction. Polymerase chain reaction (PCR) cycling was carried out as previously described.^44^ Positive (*E. coli*) and negative (no template) controls were also amplified along with sample template reactions. The amplified DNA molecules were then purified using AmpureXP paramagnetic beads (Beckman Coulter Inc., Indianapolis, IN, USA) at a final concentration of 0.8× v/v. A second, six cycle PCR was used to add a unique 12 bp barcode^45^ to each previously amplified sample using a forward primer containing the barcode + M13 forward sequence (5′-3′) and the 785 R primer (See mapping file in Table S1). The final barcoded PCR products were again cleaned using AmpureXP paramagnetic beads at a final concentration of 0.8×, quantified using the QuBit dsDNA HS assay (Life Technologies, Carlsbad, CA, USA), pooled in equimolar amounts, and concentrated to a final volume of 80 μL using two Amicon® Ultra-0.5 mL 30 K Centrifugal Filters (EMD Millipore, Billerica, MA, USA).

The final pooled library was run on the Illumina MiSeq platform (Illumina, San Diego, CA, USA) using PE250 V2 chemistry. After sequencing, reads were merged and de-multiplexed using QIIME,^46^ filtered by quality, clustered into operation taxonomic units (OTUs), and chimera checked using VSEARCH.^47^ OTU taxonomy was assigned using UCLUST^48^ and the SILVA database (Release 123; Pruesse et al. (2007)^49^). Representative sequences were aligned using pyNAST^50^ against an aligned version of the SILVA r123 database. A phylogenetic tree was created using FastTree^51^ for use in community composition analyses. Differences in community composition were estimated using weighted UniFrac indices.^52^ Sample libraries were subsampled to 12,500 reads to generate a weighted UniFrac distance matrix in order to compare microbial diversity between sites. A mapping file is included as Table S1, and the commands used to produce the final BIOM file are publicly available at https://doi.org/10.5281/zenodo.582679. Beta diversity PCoA plots were generated using the R package phyloseq,^53^ and analysis of multivariate homogeneity of group variances was tested using the PERMDISP2 test^54^ through QIIME using the R package “vegan”.^55^ Hierarchical clustering (dendrogram) was performed using the R package “clustig”.^56^ Cluster significance was also determined within this package using a similarity profile analysis (SIMPROF^57^).

Secondary alignment was produced using SILVA SINA. Post alignment OTUs were added into the r123 SILVA database using ARB.^58^ After OTUs were added into the tree, near neighbors were selected, including closely related cultivated representatives, if possible. The selected sequences were exported in FASTA format, and imported into MEGA v7.0.16.^59^ The alignment was filtered to only include the sequence region represented in OTU sequences. A maximum likelihood tree was generated using the Tamura-Nei method,^60^ and 500 bootstrap replicates. Changes in relative abundance across sample types were statistically tested using a multivariate of homogeneity of group variances test (PERMDISP2^54^).

### Data availability

After sequencing, raw reads were deposited into the NCBI sequencing read archive (SRA) under the accession number SRX2830741 (https://www.ncbi.nlm.nih.gov/sra). The mapping file for samples and corresponding barcodes can be found in Table S1.

## Electronic supplementary material

Table S1

Table S2

Calcium Carbonate Saturation (Omega)

Phylogenetic Tree of Top 25 OTUs Represented Across LHC Sample Types
