# Supplementary figures and images for: Carbonate-rich dendrolitic cones: insights into a modern analog for incipient microbialite formation, Little Hot Creek, Long Valley Caldera, California
Source: NPJ Biofilms Microbiomes. 2017 Nov 21;3:32. doi: 10.1038/s41522-017-0041-2 (PMC5698408; doi:10.1038/s41522-017-0041-2)

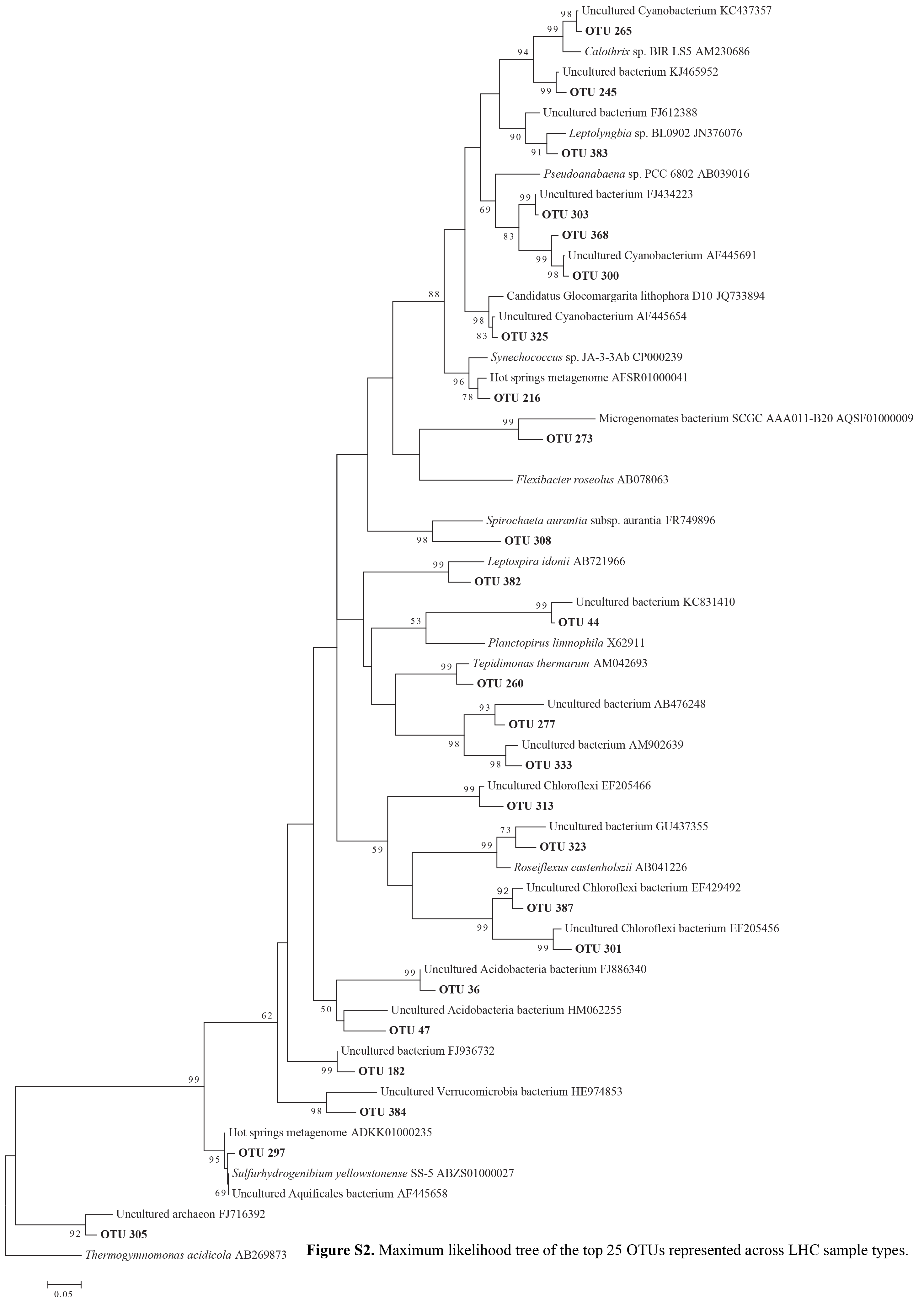

Supplement: Supplementary file 4 — Phylogenetic Tree of Top 25 OTUs Represented Across LHC Sample Types [file 41522_2017_41_MOESM4_ESM.tif]
